# Advancing ORFV‐Based Therapeutics to the Clinical Stage

**DOI:** 10.1002/rmv.70038

**Published:** 2025-05-09

**Authors:** Matthias Helmold, Ralf Amann

**Affiliations:** ^1^ Institute of Immunology University Hospital Tübingen Tübingen Germany; ^2^ Institute of Tropical Medicine University Hospital Tübingen Tübingen Germany

**Keywords:** immune stimulation, immunogenic cell death, oncolytic virus, orf virus, ORFV, parapoxvirus, vaccine, viral vector

## Abstract

The Orf virus (ORFV) is the prototype member of the parapoxvirus family and has long been recognized for its robust immunogenicity, favourable safety profile and its ability to stimulate both cellular and humoural immune responses without inducing significant anti‐vector immunity. Despite these inherent advantages, early applications of ORFV‐based technologies were limited by challenges in manufacturing scalability and uncertainties regarding clinical safety in humans. However, recent breakthroughs have transformed this therapeutic landscape. A landmark achievement is the development of Prime‐2‐CoV, an ORFV‐based anti‐COVID‐19 vaccine that has advanced into human clinical trials, providing the first clinical evidence of live ORFV's feasibility, safety and immunogenicity. This milestone, together with the establishment of a good manufacturing practice (GMP)‐compliant production process and comprehensive preclinical evaluations, has laid a robust foundation for broader clinical applications of ORFV‐based therapeutics. Moreover, the use of ORFV as an oncolytic virus therapy has shown promising results, effectively converting immunologically ‘cold’ tumours into ‘hot’ ones, underscoring its versatility as a therapeutic platform. In this review, we critically assess recent advances in ORFV‐based therapeutics, with a particular focus on vaccine development and oncolytic virotherapy (OVT). We thoroughly discuss the milestones and impact of the first ORFV‐based clinical trial, outline strategies for optimizing the technology and provide insights into overcoming remaining challenges. Collectively, these advancements position ORFV as a highly promising and versatile platform for next‐generation prophylactic and therapeutic interventions in both human and veterinary medicine, while also providing a roadmap for future innovations.

**Abbreviations**
ADCC: antibody‐dependent cellular cytotoxicity
AEs: adverse events
APCs: antigen presenting cells
ASFV: African swine fever virus
cDC1s: type 1 conventional dendritic cells
CHB: chronic hepatitis B
CRPV: cottontail rabbit papilloma virus
CSFV: classical swine fever virus
DAMPs: danger‐associated molecular patterns
DCs: dendritic cells
ERA: environmental risk assessment
GMFIs: geometric mean fold titre increase
GMO: genetically modified organism
GMP: good manufacturing practice
GMTs: geometric mean titres
GSDME: Gasdermin E
hcDNA: host cell DNA
hcProtein: host cell Protein
ICD: immunogenic cell death
ID: intradermal
IHRES: internal ribosome entry sites
IM: intramuscular
iORFV: inactivated ORFV
IP: intraperitoneal
IT: intratumoral
IV: intravenous
MVA: modified vaccinia virus Ankara
nAb: neutralizing antibody
NF‐κB: nuclear factor kappa B
NHPs: non‐human primates
NK cells: natural killer cells
OFTu cells: ovine turbinate cells
ORFV: Orf virus
OVT: oncolytic virotherapy
PAMPs: pathogen‐associated molecular patterns
PEDV: Porkine epidemic diarrhoea virus
PFU: plaque‐forming units
PRV: pseudorabies virus
RHDV: rabbit hemorrhagic disease virus
SC: subcutaneous
TAAs: tumour‐associated antigens
TFF: tangential flow filtration
TME: tumour microenvironment
TNBC: triple‐negative breast cancer
VEGF: vascular endothelial growth factor
VV: vaccinia virus
WHO: World Health Organization

## 1 Summary

Viral platforms have become a cornerstone of modern therapeutics, with diverse viruses—from adenoviruses and lentiviruses to poxviruses—being harnessed for vaccine development, gene therapy and OVT. In this rapidly evolving landscape, ORFV represents a particularly attractive candidate owing to its strong immunogenic properties, favourable safety profile and unique capacity to stimulate both innate and adaptive immunity while evading strong anti‐vector immunity. Originally known as the causative agent of contagious ecthyma in sheep and goats, ORFV's unique biological attributes—such as its strict skin tropism, cytoplasmic replication and limited host range—have spurred interest in its application as a versatile platform for therapeutic development.

Recent advances have transformed ORFV‐based technologies from an underexplored concept into a promising tool for a broad spectrum of clinical applications. Its potential extends beyond its traditional use as a paraimmune inducer to emerging roles in OVT or vaccine development, where the virus's ability to modulate the tumour microenvironment may enhance anti‐tumour immunity, and as a recombinant vaccine vector capable of delivering complex antigenic payloads. The recent clinical evaluation of Prime‐2‐CoV, an ORFV‐based SARS‐CoV‐2 vaccine—demonstrating not only the feasibility of ORFV manufacturing under GMP conditions but also confirming its safety and immunogenicity in early‐phase human trials—has provided compelling proof‐of‐concept for ORFV's clinical utility.

This review consolidates the latest advancements in ORFV‐based therapeutics, examining the fundamental characteristics that underpin its versatility and safety. It discusses how a detailed understanding of ORFV's molecular mechanisms of replication and host interaction has enabled innovative strategies in both vaccine development and oncolytic therapy. The achievements of Prime‐2‐CoV underscores the critical importance of scalable manufacturing processes and sets a new standard for the clinical translation of ORFV‐derived products. Moreover, ongoing improvements in genome engineering, immune modulation and downstream processing promise to further enhance the adaptability and efficacy of this platform.

In summary, by bridging preclinical insights with clinical achievements, this review offers a comprehensive roadmap for the future of ORFV‐based therapeutics. We explore future directions and untapped opportunities, emphasizing ORFV's potential to become a widely versatile and impactful technology in the clinical arena. Ultimately, the cumulative progress in this field addresses critical challenges in infectious diseases and oncology, paving the way for a new generation of versatile and impactful therapeutic interventions.

## 2 Characteristics and Relevance of the Orf Virus

ORFV is an enveloped virus with an ovoid morphology, measuring approximately 260 × 160 nm [1, 2]. Its genome is a linear double‐stranded DNA molecule with a high G + C content of about 64% [3, 4, 5, 6], approximately 138 kilobase pairs in length, encoding around 132 open reading frames (ORFs) [7, 8, 9]. Replication occurs entirely in the cytoplasm of the host cell, a characteristic shared with other poxviruses [10]. The viral life cycle begins with virion attachment to the host cell, followed by entry via macropinocytosis as a major pathway, although alternative routes such as clathrin‐mediated endocytosis or direct membrane fusion may also contribute depending on the host cell type and conditions [11]. Of note, the exact mechanisms governing ORFV entry remain incompletely understood and may vary across different cell types. Upon internalization, the viral core is released into the cytoplasm, where early gene transcription is initiated by the viral RNA polymerase. This is followed by DNA replication and the expression of intermediate and late genes, culminating in the assembly and release of new virions [9, 10, 12].

ORFV primarily infects sheep and goats, causing contagious ecthyma (scabby mouth), characterized by pustular lesions on the skin and mucous membranes [13]. Abrasion or broken skin is a prerequisite for ORFV to enter the body, where it replicates exclusively in regenerating epithelial keratinocytes without spreading systemically [14]. Lesions therefore stay localized and typically resolve within six to 8 weeks. Particularly in young animals, severe complications can nonetheless arise from secondary bacterial or fungal infections, making ORFV a relevant pathogen in the livestock industry causing notable economic losses [13]. While ORFV is zoonotic and can infect humans and other species, human infections are rare [13, 15], generally benign, self‐limiting and resolve within weeks without medical intervention [16, 17]. In immunocompromised patients, persistent lesions known as giant orf may develop [18], but can be effectively treated with antiviral agents like imiquimod or cidofovir [19, 20].

The immune response to ORFV infection involves a strong inflammatory reaction, triggering pro‐inflammatory cytokine production and recruitment of neutrophils, T cells, B cells and dendritic cells (DCs) to the infection site [13]. The response is primarily Type I, with Th1 cells being predominant [13, 15]. CD4^+^ T cells and interferon‐gamma (IFN‐γ) play crucial roles in viral clearance, while CD8^+^ T cells contribute to a lesser extent [13, 15]. The specific humoural response also appears to play a minor role, due to an absence of neutralizing antibodies [13, 14, 15]. Despite its initial magnitude, the immunological memory response to ORFV infection is short lived and does not confer sterilizing immunity, as hosts can be re‐infected shortly after recovery [13, 14]. Consequently, there is no approved vaccine against orf disease. This indicates a potent intrinsic immune escape mechanism most likely mediated by a multitude of ORFV encoded immunomodulatory proteins. These include five distinct gene products known to interfere at multiple segments of the nuclear factor kappa B (NF‐κB) pathway (ORF 002, 024, 073, 119 and 121) [21, 22, 23, 24, 25, 26], as well as a direct antagonist of interferon signalling mediated by the dsRNA‐dependent protein kinase PKR (ORF 020), suppressing the host cells antiviral response [27, 28]. A chemokine binding protein (ORF 112) and GM‐CSF inhibitory factor (ORF117) inhibit the recruitment of DCs and other leukocytes [29, 30, 31], while maturation and production of cytokines like tumour necrosis factor (TNF)‐α and IFN‐γ by DCs and macrophages is further suppressed by a virus encoded homologue to the anti‐inflammatory cytokine IL10 (ORF 127) [32, 33]. Furthermore, the viral VEGF gene acts as a crucial virulence factor by stimulating capillary proliferation and epithelial hyperplasia, which enhances vascular permeability and facilitates both viral replication and the development of scabs [34].

## 3 The Current Landscape of ORFV‐Based Therapeutics

The combination of strong innate immune activation and a low risk of sustained vector immunity distinguishes ORFV as a highly attractive platform for therapeutic applications. Early studies with inactivated ORFV (iORFV) have established its effectiveness as a paraimmune inducer, supporting its use in both prophylactic vaccines and OVT [35]. ORFV inherently activates key innate pathways—including TLRs, the inflammasome and the STING pathway—eliminating the need for additional adjuvants and driving robust humoural and cellular responses [36, 37]. Its replication in the cytoplasm, facilitated by prepackaged viral RNA polymerases and transcription factors, ensures efficient transgene expression and antigen presentation via MHC class I and II pathways, even in non‐permissive host [38]. Moreover, the virus's large genome permits stable integration of multiple transgenes, enabling the design of polyvalent vaccines that can target a range of pathogens and tumours [38]. With a strict skin tropism, limited systemic spread and negligible risk of genome integration, ORFV offers an excellent safety profile [39]. Crucially, the transient nature of the immune response against ORFV minimizes anti‐vector immunity, allowing for effective re‐immunization strategies. In this chapter we review the use of ORFV as an immunomodulator, as an oncolytic agent and as a vector for recombinant vaccines, highlighting its significant promise for both human and veterinary therapeutics.

### 3.1 Therapeutic Applications of ORFV as an Immunomodulator

The use of immunomodulators in clinical and veterinary medicine has a long history and iORFV has garnered significant attention for its potent immunomodulatory properties, serving historically as a paraimmune inducer. Several studies have expanded our understanding of its mechanisms and therapeutic potential, both in veterinary practice and emerging human applications [35]. Notable, the pattern of immune stimulation by iORFV is analogous to that of its active counterpart. iORFV is recognized almost immediately by the host's innate immune system primarily through antigen‐presenting cells (APCs) such as DCs and macrophages. In vitro studies have demonstrated that bone marrow‐derived conventional DCs and plasmacytoid DCs secrete high levels of interferon‐α/β upon iORFV exposure, establishing an antiviral state and activating natural killer (NK) cells for early viral control [36, 40, 41]. In addition, robust production of pro‐inflammatory cytokines—namely TNF‐α, interleukin (IL)‐12/23p40 and IL‐18—contributes to a Th1‐skewed response [42].

Historically, iORFV has been used as a paraimmune inducer in veterinary practice [35]. Its broad immunomodulatory effects have been harnessed to reduce the incidence and severity of infectious diseases, especially under stressful conditions such as crowding, where stress‐induced immunosuppression can predispose animals to secondary infections [43]. For example, studies in young calves have demonstrated that iORFV treatment can reduce the incidence of bovine respiratory disease by over 50% compared to controls while simultaneously lowering antibiotic usage—a critical advantage amid rising concerns about antimicrobial resistance [44].

In equine medicine, administration of iORFV in weaned and transported horses has been associated with a significant decrease in respiratory infections and milder clinical signs, including reduced nasal discharge, underscoring its ability to counteract stress‐related immune suppression [43]. Similar benefits have been observed in pigs, where iORFV‐based treatments have been explored for conditions such as mastitis‐metritis‐agalactia syndrome and post‐weaning diarrhoea [45, 46]. Furthermore, recent studies in dairy cattle have shown that prophylactic treatment with iORFV significantly reduced California Mastitis Test CMT scores and somatic cell counts in healthy Holstein cows, indicating that iORFV could be a viable alternative to antibiotics for protecting cattle against subclinical mastitis [47]. Moreover, immune stimulation with iORFV has shown adjuvant effects when combined with equine influenza vaccines, enhancing antibody responses against both the vaccine strain and a heterologous clade 2 strain, suggesting its suitability for improving inactivated vaccine protocols in horses and other species [48].

Recent pre‐clinical studies have expanded the potential of iORFV into human therapeutics. Its antiviral properties have been demonstrated in murine models of herpes simplex virus type 1 (HSV‐1) infection and in transgenic models of hepatitis B virus (HBV) replication [49]. In these models, low doses of iORFV achieved significant reductions in viral load, often outperforming conventional antiviral agents such as lamivudine, without inducing the adverse inflammatory responses typically associated with cytokine therapies. For instance, comparative studies have shown that iORFV derived from strains D1701 and NZ2 exhibits robust antiviral activity against HBV; notably, strain NZ2 reduced HBV DNA and core antigen levels more effectively in an HBV‐transgenic mouse model and also demonstrated superior antifibrotic effects in rat models of liver fibrosis [50].

In relation to hepatitis C virus (HCV), both iORFV strains have shown antiviral activity in vitro [50]. The induction of IFN‐α by iORFV, a cytokine central to the standard treatment for HCV, appears to suppress viral replication and may also mitigate liver fibrosis associated with chronic HCV infection. Although these findings remain preliminary, they underscore iORFV's potential as a dual therapeutic agent addressing both viral replication and liver pathology.

In a preclinical study by Korolowicz and colleagues, the novel immunomodulator AIC649—a stimulator based on the iORFV strain NZ2—was evaluated in woodchucks with chronic hepatitis B (CHB) [51]. AIC649 monotreatment induced modest reductions in serum viral DNA and hepatitis B surface and envelope antigens, exhibiting a unique biphasic response pattern. More importantly, when combined with the antiviral drug Entecavir, AIC649 markedly enhanced viral control. The combination treatment led to undetectable levels of viral antigens, elicited antiviral antibodies and correlated with increased interferon expression and suppression of viral replication in the liver. The regimen was well tolerated, with only minor and transient liver enzyme elevations, suggesting that the observed antiviral effects were based on an improvement or reconstitution of the otherwise deficient antiviral immune response typical of CHB. In 2018, AiCuris Anti‐infective Cures GmbH reported positive Phase 1 clinical trial results with AIC649 in CHB patients, confirming that a single intravenous (IV) dose was safe, well tolerated and stimulated cytokines such as IL‐1β, IL‐6, IL‐8 and IFN‐γ, while reducing IL‐10 levels [52]. These encouraging results, along with the sustained loss of viral antigens and induction of antiviral antibodies observed in woodchuck models, support the hypothesis that AIC649 can reconstitute antiviral immune responses and may lead to a functional cure in CHB patients. Furthermore, a small Phase I clinical study evaluated AIC649 in the treatment of asymptomatic or mildly symptomatic SARS‐CoV‐2 infection, although detailed results have yet to be published.

Moreover, the iORFV strain OV‐SY17 was investigated in Syrian golden hamsters against SARS‐CoV‐2 infection [53]. Treatment with iORFV significantly reduced the concentration of SARS‐CoV‐2 RNA‐containing aerosol particles, resulting in a complete blockade of aerosol transmission. This effect is likely due to decreased viral replication in both the upper and lower respiratory tracts, leading to fewer fine aerosolized viral particles. Similarly, in a guinea pig model of genital herpesvirus infection, iORFV treatment significantly reduced recurrent disease episodes, viral shedding and viral DNA load in dorsal root ganglia compared to acyclovir‐treated and placebo groups, highlighting its robust immunomodulatory and antiviral effects [54].

Furthermore, another study using the ORFV Hoping strain demonstrated its immunomodulatory and anti‐influenza properties. Although this strain was not inactivated, it stimulated human monocytes to secrete IL‐8 and TNF‐α, and conditioned medium from ORFV‐infected cells protected A549 epithelial cells from subsequent type A influenza virus infection. In murine models, pre‐exposure to ORFV via intramuscular (IM) or subcutaneous (SC) routes significantly reduced influenza virus replication, suggesting its potential as a bio‐adjuvant for influenza prevention [55].

Emerging evidence also suggests that ORFV‐mediated immunomodulation may offer anti‐fibrotic benefits. In rat models of liver fibrosis induced by carbon tetrachloride or pig serum, treatment with iORFV (particularly strain D1701) not only halted fibrosis progression but also reversed established fibrotic changes [56]. These results open new avenues for ORFV‐based therapies in managing liver fibrosis and cirrhosis.

Beyond its antiviral and antifibrotic effects, iORFV shows promise as an adjunct in cancer therapy. Pre‐clinical studies in transplantable tumour models—such as murine B16 F10 melanoma and human breast cancer xenografts—have demonstrated that ORFV treatment can slow tumour growth by enhancing NK cell cytotoxicity and increasing IFN‐γ secretion. Even in immunocompromised models, where conventional immune responses are diminished, ORFV administration yielded measurable antitumour effects, suggesting its potential to complement existing cancer therapies [57].

In summary, the diverse preclinical studies reviewed here underscore the versatility of iORFV as an immunomodulatory agent. Its ability to trigger robust Type I immunity and capacity to reduce viral loads, enhance vaccine responses and mitigate inflammatory and fibrotic processes in both veterinary and human models supports its value as a paraimmune inducer.

### 3.2 Using ORFV as an Oncolytic Virus for the Treatment of Tumours

OVT has gained significant attention as a cancer immunotherapy approach, utilizing genetically engineered or naturally occurring viruses to selectively infect tumour cells [58]. Effective OVT relies on two fundamental principles: direct tumour lysis and immune‐mediated tumour clearance [59]. While viral replication within tumour cells contributes to immediate tumour reduction, long‐term therapeutic success is largely driven by the virus's ability to reshape the tumour microenvironment (TME) and elicit systemic antitumour immunity [60]. In this way, effective oncolytic viruses not only kill tumour cells but also reshape the immune landscape, driving both innate and adaptive responses that contribute to the elimination of residual tumours beyond sites of viral replication [58, 59, 61]. The induction of immunogenic cell death (ICD) plays a particularly critical role in this process, as dying tumour cells release danger‐associated molecular patterns (DAMPs) and tumour‐associated antigens (TAAs) that enhance antigen presentation and immune activation [62, 63]. This response is further amplified by Type I interferons (IFN‐I), which enhance DC maturation, recruit NK cells, promote CD8 T cell expansion. Additionally, IFN‐I repolarizes immunosuppressive M2 macrophages, which are typically associated with tumour progression, into a pro‐inflammatory M1 phenotype, thereby reinforcing antitumour immunity and TME remodelling [64, 65]. Thus, an optimal oncolytic virus must not only exhibit efficient tumour tropism but also elicit a strong Type I immune response, effectively transforming immunosuppressive ‘cold’ tumours into ‘hot’ tumours primed for immunotherapy [66].

Among various viral platforms explored [58], poxviruses, particularly vaccinia virus (VV) [67], have been widely studied due to their efficient replication in tumour cells, potent immunostimulatory effects and suitability for genetic modifications that enable the expression of therapeutic transgenes, further enhancing their oncolytic and immune‐modulatory potential. More recently, ORFV has emerged as a promising candidate for OVT. Early studies on iORFV demonstrated anti‐tumour effects against mouse malignant melanoma and human breast cancer cells, in which NK cells and cytokines (IFN‐γ and IL‐12) play important roles [57]. However, these formulations lacked replicative capacity and direct oncolytic activity.

The seminal study by Rintoul, Lemay, Tai, and et al. [68] marked a turning point, demonstrating for the first time that a replication‐competent ORFV not only effectively reduces tumour burden in both immunocompetent murine models and human xenografts, but also induces potent immune activation. The study delineates how ORFV activates DCs, which in turn facilitate NK cell activation. This DC‐NK interplay is crucial as it suggests that NK cells may be activated indirectly by DCs, providing a detailed look into the immune system’s dynamics in response to ORFV. In exploring the activation of NK cells, Rintoul et al. detail that ORFV stimulates both primary subsets: cytokine‐producing but poorly cytotoxic NK cells, and highly cytotoxic but less cytokine‐productive NK cells. This dual activation mode enhances the antitumour immune response by combining direct cellular cytotoxicity with cytokine‐driven immune modulation [69], supporting a Th‐1 dominated response essential for effective antitumour activity.

Following this initial study, the contribution of NK cells to ORFV‐mediated tumour control has been validated in other tumour models. Van Vloten et al. [70] demonstrated that in an advanced ovarian cancer setting, oncolytic ORFV—administered either IV or intraperitoneally (IP)—triggered potent NK cell activation, which in turn was essential for immediate antitumour effects. Of note, they showed that NK cells worked in tandem with type 1 conventional dendritic cells (cDC1s): depletion of either NK cells or cDC1s largely abolished ORFV's antitumour efficacy. This highlights that ORFV‐induced signals may engage not only NK cells directly but also drive a ‘help’ signal from cDC1s, which is of significant importance as NK:cDC1 interactions is correlated with increased overall survival in numerous cancer types [71]. Beyond ovarian cancer, Choi et al. [72] illustrated how a chimeric parapoxvirus (CF189, closely related to ORFV) significantly reduced metastatic triple‐negative breast cancer (TNBC) burden in mice by recruiting NK cells to the periphery of tumour lesions. Interestingly, NK cells were found infiltrating treated tumours early, suggesting that NK cell–mediated cytotoxicity might be an initial wave of tumour clearance before additional adaptive responses come into play. While NK cells appear to be first‐line responders to ORFV infection, other immune compartments also contribute to antitumour immunity. Recent investigations highlight the role of neutrophils as important mediators in ORFV‐driven immune responses. Minott, van Vloten, Chan et al. [73] used a murine pulmonary melanoma metastasis model to show that oncolytic ORFV causes an early and massive infiltration of neutrophils that paradoxically promotes, rather than hinders, therapeutic viral spread. Typically, virus‐induced neutrophils can limit viral replication [74], but here they correlated with reduced tumour burden and enhanced ORFV amplification. In a follow‐up study [75], they examined immune responses over time in an ovarian cancer model and described a dynamic interplay in which neutrophils rapidly infiltrate the ascites, followed by activation of NK cells, T cells, and cDC1s. This orchestrated recruitment strongly correlated with improved tumour control, underscoring the importance of sequential immune cell infiltration for durable antitumour efficacy. Adaptive immunity also plays a crucial, though sometimes context‐dependent, role. Van Vloten et al. [70] reported that tumour‐targeting antibodies form in ORFV‐treated mice, leading to NK cell–mediated antibody‐dependent cellular cytotoxicity (ADCC), a mechanism that promotes the targeted elimination of tumour cells [76]. Their data suggest that robust antibody responses targeting multiple tumour antigens might extend antitumour protection and reduce the likelihood of immune evasion by single‐antigen loss. In that same study, CD8+ T cells were dispensable for the primary antitumour effect yet contributed to longer‐term tumour control, especially when NK cells and cDC1s were intact. R. Wang, Mo et al. [77] similarly noted that ORFV infection leads to the release of DAMPs, such as HMGB1, facilitating DC maturation and antigen presentation. These collective findings illustrate how ORFV, through iterative cross‐talks between virus‐mediated cytolysis and immune amplification, can transform an immunosuppressive TME into one rich in both innate (NK cells, neutrophils) and adaptive (T and B cells) effectors—key to controlling established and residual disease [60].

Various studies have examined the direct cytopathic activity of ORFV on tumour cells, highlighting a multifaceted killing process that integrates classical apoptosis, cell‐cycle arrest and—most intriguingly—strongly immunogenic cell death. Several groups show that ORFV infection frequently induces a G2/M arrest and subsequently caspase‐dependent apoptosis [77, 78, 79]. For instance, R. Wang, Mo et al. [77] reported that in human lung cancer and murine LLC cells, ORFV disrupts cell‐cycle checkpoints by downregulating cyclin B1, culminating in apoptosis. Deng et al. [78] observed similar mechanisms in breast cancer models, where cyclin E and CDK2 levels drop upon infection, linking cell‐cycle blockade to pro‐apoptotic pathways. While these findings broadly agree on apoptosis as a key route, some authors note differences in ORFV's replicative capacity and direct oncolysis across cell lines, indicating that direct oncolysis may vary depending on tumour‐intrinsic factors such as interferon pathway defects or Ras‐driven proliferation [70, 78].

Notably, emerging data suggest that ORFV can also trigger forms of ICD that provoke robust immune activation. Among these, pyroptosis has garnered particular attention [80]. Pyroptotic cell death involves the activation of gasdermins, pore‐forming proteins that cause cell swelling, lysis and the release of proinflammatory signals [81]. J. Lin, Sun et al. [82] demonstrated that ORFV drives Gasdermin E (GSDME)‐mediated pyroptosis by stabilizing GSDME protein levels via reduced ubiquitination, thereby enabling caspase‐3 to cleave GSDME and induce a highly inflammatory form of cell death. This pyroptotic process can override the typical ‘silent’ apoptosis in GSDME‐low tumours, fuelling a potent local immune response. While the extent of pyroptosis varies among different tumour models, most authors agree that it constitutes a particularly immunogenic mechanism of tumour cell death [83, 84, 85]. Another ICD pathway implicated in ORFV's oncolytic arsenal is autophagy [86]. Huang et al. [87] reported that in nasopharyngeal carcinoma cells, ORFV infection suppresses the AKT/mTOR axis, upregulating LC3‐II and facilitating autophagic flux. This leads to tumour cell destruction accompanied by the release of DAMPs, helping recruit and activate immune cells. Although some groups debate whether autophagy primarily supports or hinders ORFV replication [70, 87], the consensus is that ORFV‐induced autophagy can contribute to cell death and further immunostimulation. Taken together, these findings illustrate how ORFV leverages multiple overlapping killing modalities—apoptosis, pyroptosis, autophagy—that not only destroy tumour cells but also stimulate local immune responses.

Although practical experience with ORFV‐based combination regimens remains scarce, growing evidence suggests that pairing ORFV with conventional treatments can improve therapeutic outcomes. By enhancing tumour immunogenicity and targeting residual disease, ORFV may complement standard approaches such as surgery or targeted therapy. For instance, Van Vloten et al. [70] reported that perioperative ORFV administration reduced metastasis and ascites formation in ovarian cancer models following cytoreductive surgery, underscoring how localized immunostimulation can eliminate residual tumour cells after the primary mass is resected. More controversial is ORFV's synergy with checkpoint blockade: while J. Lin, Sun et al. [82] showed that ORFV effectively ‘warms up’ cold tumours and sensitizes them to anti–PD‐1 therapy, Van Vloten et al. [70] found minimal benefits in their model—possibly due to low neoantigen expression or suboptimal timing. Beyond immunotherapy, Deng et al. [78] demonstrated that combining ORFV with a PAK4 inhibitor synergistically slows breast cancer progression, hinting at broader opportunities to merge the virus with targeted agents.

Another practical consideration is the lack of potent neutralizing antibodies to ORFV [13, 14, 15], which allows multiple rounds of viral administration without substantially compromising efficacy. Such iterative dosing regimens are particularly appealing in tumours that display heterogeneous or evolving resistance to single‐agent therapies.

**FIGURE 1:**
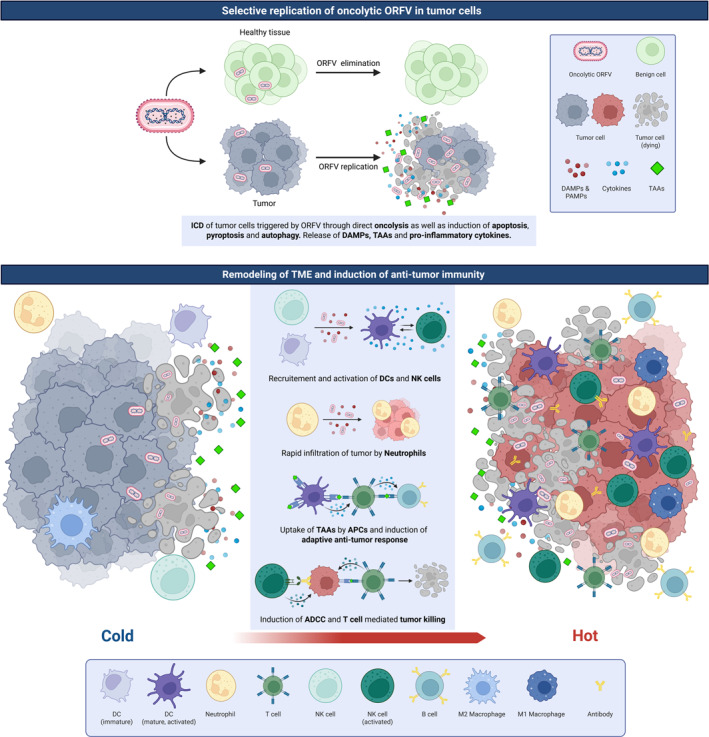
Anti‐tumour mechanisms of oncolytic ORFV. Oncolytic ORFV selectively replicates in tumour cells, inducing highly immunogenic cell death through direct lysis as well as the induction of apoptosis, pyroptosis and autophagy, while sparing healthy cells. The release of viral progeny, TAAs and DAMPs or pathogen‐associated molecular patterns (PAMPs) promotes the recruitment and activation of innate immune cells. This leads to the release of pro‐inflammatory cytokines, further enhancing immune activation. As a result, APCs prime a robust humoural and cellular adaptive anti‐tumour response, ultimately transforming the TME from immunologically inert (‘cold’) to highly reactive (‘hot’), facilitating the effective clearance of malignant cells.

In summary, ORFV has emerged as an oncolytic agent that combines broad tumour tropism with a favourable safety profile, as its infections in humans are typically localized and self‐limiting. By inducing a highly immunogenic form of cell death, along with robust innate and adaptive responses, ORFV can convert immunologically inert tumours into inflamed sites that respond more effectively to therapy as summarized in Figure 1. Moreover, ORFV's minimal susceptibility to neutralizing antibodies enables repeated administration, whereas its genome can be engineered to deliver targeted therapeutic payloads. Nonetheless, critical gaps remain, and translating ORFV‐based approaches into clinical practice requires a deeper understanding of viral replication dynamics, optimal patient selection, and possible synergies with existing treatments—such as surgery, targeted inhibitors, or checkpoint blockade. Particular attention must be paid to ensuring that tumours are permissive for ORFV replication and that the patient's immune status supports a robust antiviral and antitumour response. Addressing these considerations in preclinical and early‐phase clinical studies will be critical for harnessing ORFV's full potential. Once these parameters are clarified, ORFV could profoundly enhance the landscape of OVT by combining direct tumour lysis with a potent, adaptable immune response.

### 3.3 Using ORFV as a Vector for the Development of Recombinant Vaccines

Over the past decades, two main ORFV strains have been employed for the development of recombinant vaccines: D1701 and IA82. While IA82 has been used exclusively in the veterinary field, D1701‐based vaccines have been successfully applied for both human and veterinary vaccine development.

#### 3.3.1 Characteristics and Advantages of IA82‐Based Vaccines

ORFV strain IA82 originates from a field isolate obtained in 1982 from the nasal secretions of a lamb at the Iowa Ram Test Station, USA and was subsequently passaged in ovine foetal turbinate cells [88]. The strain retained its virulence in the natural host and has therefore been repeatedly employed to explore the roles of various encoded virulence factors by using knock‐out recombinants in vivo in sheep [26, 88, 89].

Investigations into its potential as a recombinant vaccine vector, particularly for veterinary applications, have been conducted by Diel et al. Initial efforts demonstrated that transgenes can be stably integrated in place of the non‐essential NF‐κB inhibitors ORF 121 and ORF 024 and expressed using the established VV7.5 early/late promoter [90]. Prime‐boost immunization of pigs and cattle with recombinants expressing the rabies glycoprotein from either locus induced strong neutralizing antibody (nAb) responses in both species, demonstrating the immunogenicity of this vector even in non‐permissive hosts and, for the first time, in cattle. Notably, significantly higher antibody titres were achieved when the antigen was integrated in place of ORF 121 rather than ORF 024, indicating a beneficial effect of this knockout on the vector's immunogenicity and establishing this integration site for subsequent studies [90].

An IA82^Δ121^ recombinant expressing the spike protein of porcine epidemic diarrhoea virus (PEDV) induced robust, specific IgG, IgA and nAb responses in pigs following three IM immunizations. This not only resulted in protection against clinical symptoms and reduced viral shedding in vaccinated animals after challenge [91], but was in a later study also found to confer passive immunity to their offspring. Neutralizing antibodies, as well as PEDV‐specific IgG and IgA, were detected in the serum of piglets born to gilts immunized during pregnancy, resulting in reduced morbidity and mortality after challenge and demonstrating passive transfer through colostrum and milk [92]. Given the high susceptibility of piglets to PEDV, this represents a major contribution to efficient disease control that is largely unmet by currently available inactivated or subunit vaccines [93].

Efforts to employ ORFV for the control of Influenza A viruses of swine (IAV‐S) demonstrated the induction of potent, specific cellular and humoural responses by an IA82^Δ121Δ127^ vector encoding both the haemagglutinin (HA) and nucleoprotein (NP) of the prevalent H1N1 strain [94]. In direct comparison to a recombinant encoding only HA, the additional integration of the NP protein in place of the non‐essential virulence factor ORF127 (IL‐10 homologue) increased both CD8+ T cell and nAb responses, while shifting the CD4+ response from a Th2 to a Th1 bias, leading to enhanced protection against IAV‐S challenge. Notably, serum antibodies also exhibited broader cross‐neutralization activity against a panel of contemporary IAV‐S isolates representing the major genetic clades circulating in swine, highlighting the vector's potential for designing broadly protective multivalent vaccines against pathogens with high antigenic diversity. Subsequent studies using consensus or chimeric HA proteins confirmed the induction of humoural and cellular responses protecting against challenge with divergent IAV‐S strains [95, 96]. Here, cross‐reactive IgG and IgA were detected in the bronchoalveolar lavage of vaccinated animals, indicating that ORFV‐based vaccines are capable of mediating mucosal immunity [95].

In addition, IA82 ^Δ121^ encoding the African swine fever virus (ASFV) p30 protein induced a strong humoural response in pigs, comparable to that seen in natural ASFV infection. The induced antibodies were shown to mediate antibody dependent cellular cytotoxicity, potentially contributing to protection against ASFV by aiding in the clearance of infected cells [97].

Taken together, these studies demonstrate the potential of IA82‐based vaccines to effectively combat relevant infectious diseases in multiple important and non‐permissive livestock species by safely inducing potent humoural and cellular responses against encoded foreign antigens.

#### 3.3.2 Characteristics and Advantages of the D1701‐VrV Strain

The attenuated D1701‐V strain was derived from an ORFV isolate obtained from a lamb in Düsseldorf, Germany, in 1972. After successive passages in ovine and bovine cell cultures and adaptation to the African green monkey kidney cell line Vero, the virus became highly attenuated, showing asymptomatic progression even in immunosuppressed sheep [9]. Further attenuation was achieved by deleting the major virulence factor VEGF‐E [98, 99], resulting in the D1701‐VrV strain used in our studies. This strain allows for the stable integration of heterologous transgenes at specific genomic loci, such as the original VEGF‐E location (VEGF locus), with expression driven by the native P_vegf or synthetic P1 and P2 early promoters [100, 101].

The D1701‐VrV strain of ORFV exhibits unique characteristics that make it an attractive platform for vaccine development. A key advantage of this strain is its ability to induce strong immune responses without the need for additional adjuvants. This inherent immunostimulatory property enables D1701‐VrV to effectively activate both humoural and cellular immune pathways, which are essential for achieving protective vaccine efficacy.

Multiple studies have demonstrated the potent immunogenicity of D1701‐VrV‐based vaccines across various animal models. For instance, a single vaccination with a D1701‐VrV recombinant expressing the rabies glycoprotein induced sustained high titres of neutralizing antibodies in cats, dogs and mice, conferring protection against high‐dose intracranial challenge with the rabies virus in mice [101]. In pigs, D1701‐VrV vaccines expressing antigens from pseudorabies virus (PRV) or classical swine fever virus (CSFV) elicited protective immunity [102, 103]. Moreover, vaccination of rabbits with D1701‐VrV expressing the rabbit hemorrhagic disease virus (RHDV) antigen resulted in protection against lethal challenge, highlighting the vector’s efficacy in inducing robust humoural responses [104].

Beyond humoural immunity, D1701‐VrV is also effective in eliciting strong cellular immune responses. In the cottontail rabbit papilloma virus (CRPV) model, repeated vaccination with D1701‐VrV recombinants expressing CRPV epitopes led to a significant reduction in tumour growth in 84% of animals, mediated by specific cellular responses [105]. Importantly, the vector induces antigen‐specific CD4^+^ and CD8^+^ T cell responses without generating significant responses against vector‐derived epitopes, indicating negligible anti‐vector immunity [106]. This feature allows for repeated booster vaccinations to enhance transgene‐specific immune responses, even in individuals previously exposed to ORFV [107].

**FIGURE 2:**
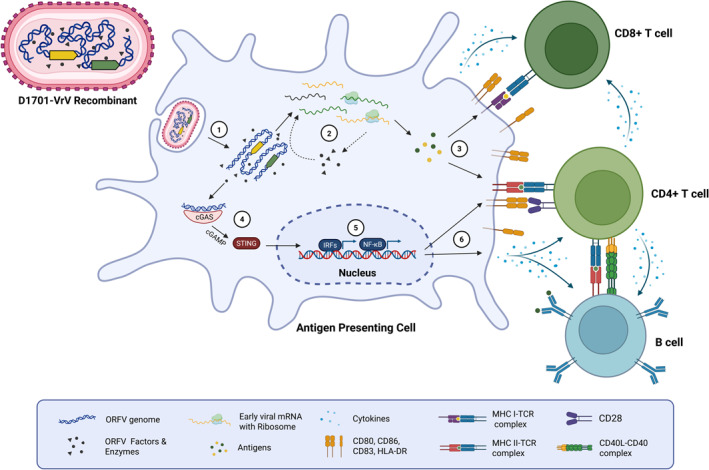
Mode of action of ORFV D1701‐VrV based vaccines. Upon administration, D1701‐VrV recombinants encoding heterologous antigens (also called transgenes; depicted as yellow and green arrows) under the control of synthetic or native early‐phase promoters are internalized by professional APCs, such as macrophages and DCs, via receptor‐independent macropinocytosis (1). Inside the cytoplasm, the viral core is uncoated, releasing the genome, which is transcribed by viral RNA polymerases and transcription factors prepackaged within the particle (2). This early‐phase transcription includes the encoded transgenes and essential viral factors for sustained expression. Importantly, gene expression remains restricted to the early phase, preventing progression of the poxviral infection cycle to genome replication or late‐phase expression. Expressed transgenes are processed by the proteasome and presented on MHC class I and II molecules, activating CD8+ and CD4+ T cells (3). Concurrently, recognition of the viral double‐stranded DNA genome by the cGAS‐STING pathway (4) triggers innate immune signalling, leading to the activation of interferon regulatory factors (IRF) and NF‐kB (5). This activation drives the release of cytokines, such as IL‐12 and CXCL10, and the upregulation of costimulatory molecules, including CD80, CD86 and OX40L (6). The combination of antigen presentation and APC activation facilitates robust stimulation of adaptive and innate immune responses, including the activation of CD4^+^ and CD8^+^ T cells, B cells and NK cells. This results in potent humoural and cellular immunity against the encoded transgenes.

Recent analyses have shown that the immune responses elicited by D1701‐VrV critically depend on its uptake by APCs, where heterologous antigens are expressed and presented. Activation of these APCs is further enhanced by innate immune signalling pathways, such as the STING pathway, which promotes leucocyte activation and the induction of adaptive immune responses [37, 108, 109]. This mechanism is illustrated in Figure 2. In line with the complete replication deficiency of D1701‐VrV in vivo [39], our studies strongly indicate that viral gene expression in APCs is limited to the early phase of poxviral replication (unpublished data). Its replication is restricted to the cytoplasm of infected cells, eliminating the risk of integration into the host genome and oncogenic potential [9, 110]. Additionally, D1701‐VrV exhibits a strict skin tropism with no evidence of systemic spread [39], while its specific targeting of APCs reduces the likelihood of off‐target effects [37]. Importantly, D1701‐VrV is replication‐deficient in vivo, minimizing the risk of uncontrolled viral spread [39].

In terms of manufacturing and stability, D1701‐VrV‐based vaccines can be efficiently produced in suspension HEK293 cells, enabling large‐scale production in bioreactors with optimized parameters to maximize virus yield [111]. The downstream processing includes effective clarification and purification steps, ensuring high purity of the final product [112, 113]. Notably, D1701‐VrV demonstrates excellent stability, with a shelf‐life exceeding 2 years when stored at 4°C or frozen. Its inherent resistance to drying and freezing suggests the feasibility of developing a lyophilized vaccine product, which would be advantageous for distribution and storage, especially in regions with limited cold chain infrastructure [114, 115, 116].

Other advantages of D1701‐VrV include its high genomic capacity, allowing for the stable integration of multiple large transgenes. This enables the development of polyvalent vaccines expressing several antigens simultaneously, as well as the inclusion of immunomodulatory elements like cytokines or costimulatory molecules to further enhance immunogenicity [100, 110]. The vector’s versatility and rapid prototyping capabilities are critical during emerging infectious disease outbreaks, allowing for swift generation and testing of vaccine candidates. Collectively, these characteristics—demonstrated strong immunogenicity, favourable safety and tolerability, efficient manufacturing, stability and versatility—position D1701‐VrV as a promising platform for vaccine development against a wide range of infectious diseases. Its ability to induce potent immune responses without significant anti‐vector immunity addresses many limitations observed with other vaccine platforms and supports its potential for both prophylactic and therapeutic applications.

## 4 Prime‐2‐CoV: The First ORFV‐Based Therapeutic Entering the Clinic

### 4.1 Preclinical Development of Prime‐2‐CoV

#### 4.1.1 Scientific Rationale and Vaccine Design

**FIGURE 3:**
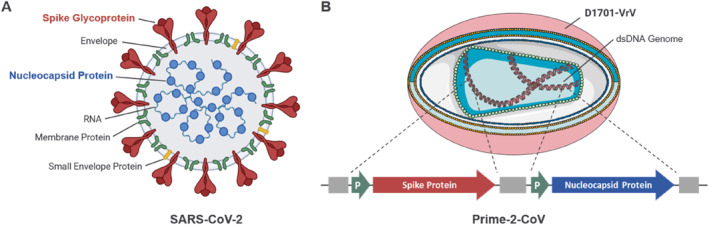
Design of Prime‐2‐CoV as a multi‐antigenic vaccine against SARS‐CoV‐2. (A) Structure of the SARS‐CoV‐2 virion. (B) Illustration of Prime‐2‐CoV, designed to express both the spike and nucleocapsid antigen of SARS‐CoV‐2 from two separate loci within the genome of the D1701‐VrV vector under control of viral early‐phase promoters (P).

Prime‐2‐CoV was designed as a multi‐antigenic vaccine candidate that expresses both the Spike protein and the Nucleocapsid protein of SARS‐CoV‐2 (Figure 3). The Spike protein is presented in a prefusion‐stabilized form which has been shown to enhance vaccine immunogenicity and efficacy [117]. Prime‐2‐CoV_Beta includes mutations specific to the Beta variant of SARS‐CoV‐2, enhancing its relevance against more actual variants of concern [117]. The inclusion of the Nucleocapsid protein, which is highly conserved among different SARS‐CoV‐2 variants, aims to induce a broader and more robust immune response [118, 119, 120]. Several vaccine candidates targeting the Nucleocapsid protein have been evaluated in preclinical settings [38, 121, 122, 123, 124], advanced into clinical development [125, 126, 127] and have shown that combining Spike and Nucleocapsid antigens can improve vaccine efficacy [121, 122, 123] by substantially enhancing cell‐mediated immunity and facilitating cross‐reactive T cell responses. This approach could potentially provide protection independent of the frequently mutating Spike protein, addressing challenges posed by emerging variants [120, 128, 129, 130, 131].

#### 4.1.2 Preclinical Safety Evaluation

Prime‐2‐CoV underwent extensive preclinical evaluation to ensure both safety and minimal environmental impact [39]. Biodistribution studies in rats and immunodeficient mice vaccinated either IM or IV demonstrated that the ORFV vector primarily remains at the injection site, with minimal presence in other organs that rapidly declines over time. Importantly, no evidence of viral replication was detected, confirming the replication‐deficient nature of the vector. This characteristic is crucial for safety, reducing the risk of unintended spread and increasing suitability for use in vulnerable populations [132]. Furthermore, shedding studies conducted in rabbits and rats revealed no detectable release of the vaccine vector in bodily fluids such as urine, feces or saliva, even when rats were administered a full human dose. These findings further support the vaccine’s favourable safety profile, particularly regarding environmental risks [133]. In addition, Prime‐2‐CoV underwent an environmental risk assessment (ERA), which confirmed that the recombinant ORFV vector poses negligible risks as a genetically modified organism (GMO). No additional risk mitigation strategies were deemed necessary, emphasizing its environmental safety [39].

#### 4.1.3 Preclinical Immunogenicity and Efficacy

The immunogenicity and protective efficacy of Prime‐2‐CoV were evaluated in various animal models [38, 134]. In mice, vaccination induced a pronounced Type 1 immune response, with the generation of antibodies and T cells against both antigens, although responses against the Spike protein were stronger. Importantly, compared to an ORFV‐based vaccine expressing only the Spike protein (ORFV‐S), the co‐expression of the Nucleocapsid protein did not impair the Spike‐specific immune response [38]. This finding suggests that the inclusion of a second antigen does not negatively impact the immune response against the other antigen, an important conclusion for the design of ORFV‐based multi‐antigenic vaccines [38].

In hamsters, immunization with Prime‐2‐CoV conferred complete protection of both the upper and lower respiratory tracts against SARS‐CoV‐2, whereas the protective effect in animals vaccinated with ORFV‐S was limited to the lower respiratory tract. This finding supports the benefit of the multi‐antigenic vaccine design [38]. Additionally, Burri et al. demonstrated that Prime‐2‐CoV induced cross‐neutralizing antibodies against multiple SARS‐CoV‐2 strains, including D614G, Delta and Omicron BA.1, mediating cross‐protection against different variants [134].

In non‐human primates (NHPs), Prime‐2‐CoV showed strong immunogenicity and protective efficacy. Rhesus macaques developed humoural immune responses against the Spike protein and T cell responses against both Spike and Nucleocapsid proteins after two vaccinations. Following SARS‐CoV‐2 infection, vaccinated animals exhibited rapid viral clearance in the nose and throat and no detectable virus in bronchoalveolar lavage fluid, indicating that the virus remained restricted to the upper airways without affecting the lungs. A long‐term experiment confirmed the durability of protection over a period exceeding 5 months after two doses of 3 × 10^7^ PFU of Prime‐2‐CoV, with 80% of primates protected, in contrast to none in the control group [38].

The ability of Prime‐2‐CoV to boost existing memory responses after SARS‐CoV‐2 infection was also investigated. Vaccination following infection elicited high titres of Spike‐binding antibodies and potent virus‐neutralizing capacity, as well as a more consistent increase in Nucleocapsid‐specific IgG levels compared to re‐infection. Furthermore, experiments testing Prime‐2‐CoV in heterologous combinations with approved COVID‐19 vaccines—based on mRNA (Comirnaty, BioNTech), protein subunit (Nuvaxovid, Novavax), viral vector‐based (Vaxzevria, AstraZeneca and Jcovden, Johnson & Johnson) or inactivated vaccine (VLA2001, Valneva)—corroborated that Prime‐2‐CoV is well‐suited for heterologous prime‐boost regimens. These combinations resulted in enhanced humoural and cellular immune responses compared to homologous vaccination with the respective vaccines. Considering that a large portion of the global population has been infected with SARS‐CoV‐2 and/or vaccinated with an approved COVID‐19 vaccine, these findings support the potential of Prime‐2‐CoV as an effective booster against COVID‐19.

### 4.2 Clinical Evaluation of Prime‐2‐CoV

Building on these promising safety and immunogenicity data Prime‐2‐CoV proceeded to Phase I clinical evaluation. Prime‐2‐CoV was evaluated in two Phase I clinical studies—ORFEUS and UKT—to assess its safety, reactogenicity, and immunogenicity in healthy individuals who had previously received approved mRNA vaccines [135, 136]. Both studies shared similar designs focused on dose‐finding and safety assessments. The inclusion and exclusion criteria were largely comparable, ensuring consistency in participant selection.

#### 4.2.1 Study Designs and Participant Demographics

The *ORFEUS* study was a Phase I, multi‐centre, open‐label, dose‐escalation trial conducted in Germany and the United States [135]. It enrolled 72 participants, including 48 younger adults aged 18–55 years and 24 older adults aged 65–85 years, who had received at least two prior mRNA vaccinations. Participants were assigned to four dose groups—3 × 10^5^, 3 × 10^6^, 1.5 × 10^7^ and 3 × 10^7^ plaque‐forming units (PFU)—and received two IM doses of Prime‐2‐CoV_Beta on days 1 and 29. The study initially aimed to include SARS‐CoV‐2 vaccine‐naïve individuals, but this goal was largely unmet due to the widespread rollout of authorized vaccines during the study period.

The *UKT* study was a Phase I, multi‐centre, open‐label trial in Germany with 60 participants aged 18–55 years who had received at least three prior mRNA vaccinations [136]. Participants received a single IM dose of Prime‐2‐CoV in an escalated dose regimen, testing the same four dose levels as the ORFEUS study, with the addition of a lower dose group of 3 × 10^4^ PFU.

Both studies utilized standardized assays for immunogenicity assessments to ensure comparability of results. Serological and immunogenicity analyses were conducted at Vismederi Labs, a CEPI‐accredited laboratory, to ensure consistency and comparability of results across both studies.

#### 4.2.2 Safety and Reactogenicity

Both studies demonstrated that Prime‐2‐CoV_Beta was generally safe and well‐tolerated. The most common adverse events (AEs) reported were pain at the injection site, headache and fatigue. These reactions were predominantly mild to moderate in intensity and typically resolved within a few days. Severe AEs were rare and not directly attributed to the vaccine. Importantly, no serious AEs related to Prime‐2‐CoV_Beta were observed in either study, and none of the prespecified stopping rules were triggered.

Reactogenicity was similar across dose groups. Most solicited AEs occurred within the first 2 days post‐vaccination and had a short duration, with median times to resolution of one to 2 days. The distribution of AEs was similar across different dose cohorts and did not show a clear dose‐response relationship. However, a trend toward a higher total incidence of solicited local and systemic AEs was noted for the two highest dose cohorts, although this increase was not attributed to specific symptoms but rather the overall incidence.

Solicited systemic reactions were more common among younger participants than older ones, consistent with reports from other COVID‐19 vaccine studies [137, 138]. Frequencies of solicited reactions were generally similar following the first and second vaccinations. The profile of reported reactions is comparable to other COVID‐19 vaccines such as mRNA vaccines [138, 139] or viral vector‐based vaccines [125, 140, 141], but appeared to be milder and less frequent at the tested doses.

#### 4.2.3 Immunogenicity

Prime‐2‐CoV_Beta elicited robust humoural and cellular immune responses, particularly at higher doses. In the ORFEUS study, participants in the highest dose group (3 × 10^7^ PFU) showed significant increases in neutralizing antibody titres against multiple SARS‐CoV‐2 variants, including the ancestral strain and Beta, Delta and Omicron BA.5 variants. Geometric mean titres (GMTs) increased by 2‐ to 3.7‐fold 28 days after the first vaccination and were maintained through day 183. The second dose did not significantly boost antibody levels, suggesting a plateau in the humoural response.

Older adults exhibited lower immune responses compared to younger participants, likely due to immunosenescence [142, 143]. However, the vaccine still induced measurable antibody levels in this group.

The UKT study showed similar trends, with the highest dose cohort demonstrating significant antibody responses against all tested variants. GMT increases were most pronounced against the Beta and Omicron BA.5 variants, with a 2.1‐fold increase. The single‐dose regimen resulted in declining antibody levels over time, indicating the potential benefit of a second dose as observed in the ORFEUS study.

Prime‐2‐CoV_Beta also induced strong cellular immunity. In the ORFEUS study, participants in the highest dose group developed robust, polyfunctional CD4^+^ Th1 and CD8^+^ T cell responses, which were enhanced after the second dose. This highlights the vaccine’s capacity to induce durable cellular immunity, which is critical for long‐term protection.

Binding antibody responses against the S1 subunit, and receptor‐binding domain of the Spike protein were also significantly enhanced in higher dose cohorts in both studies. Importantly, the vaccine induced significant antibody responses against the Nucleocapsid protein, which is not targeted by mRNA vaccines. The response to the Nucleocapsid protein was increased by 6.8‐fold and 5.1‐fold in the 3 × 10^7^ PFU groups of the ORFEUS and UKT studies, respectively. Generally, the increases in GMTs, excluding the nucleocapsid titres, were noticeably lower in the UKT study compared to the ORFEUS study. This difference is likely due to elevated baseline titres in UKT participants, possibly resulting from the additional mRNA vaccinations they received.

#### 4.2.4 Interpretation and Comparison With Other SARS‐CoV‐2 Vaccines

Interpreting the immune responses induced by Prime‐2‐CoV is complex due to several factors inherent to the study conditions. Conducted during the COVID‐19 pandemic, both studies involved participants with highly variable immune statuses arising from previous mRNA vaccinations and natural SARS‐CoV‐2 infections. This often resulted in hybrid immunity against multiple variants, leading to exceptionally high baseline levels of humoural immunity and complicating the assessment of Prime‐2‐CoV’s immunogenicity. In fact, the ORFEUS study observed a negative correlation between pre‐existing antibody titres and the boosting effect of Prime‐2‐CoV. Participants with high baseline immunity experienced limited increases in neutralizing antibodies, suggesting a ‘ceiling effect’. This aligns with findings from other booster vaccine studies, indicating that individuals with high pre‐existing immunity may have diminished responses to additional doses [144, 145, 146]. Additionally, the absence of a control group vaccinated with an approved COVID‐19 vaccine further challenges direct comparisons of immunogenicity profiles.

When compared to other vaccine studies, Prime‐2‐CoV’s neutralizing antibody responses were promising. For instance, a study using the protein‐based NVX‐CoV2373 (Novavax) vaccine with a comparable study design as the UKT study reported a 1.75‐fold increase against the original SARS‐CoV‐2 and a 1.58‐fold increase against the BA.5 variant [147]. In contrast, Prime‐2‐CoV achieved a 1.41‐fold increase against the original virus and a 2.11‐fold increase against BA.5 in the UKT study, indicating comparable responses against certain variants. However, Moderna’s mRNA1273 vaccine demonstrated higher fold‐increases in a comparable study setting, with a 3.11‐fold increase against the original virus and a 3.78‐fold increase against the BA.5 variant [148]. When comparing responses to the nucleocapsid protein, Prime‐2‐CoV induced slightly higher geometric mean fold titre increase (GMFIs) after a single immunization compared to a bivalent modified vaccinia virus Ankara (MVA)‐based vaccine (5‐fold vs. 2‐ to 4.5‐fold GMFI) [125].

Anti‐ORFV antibodies were measured to assess the immune response against the viral vector. While no responses were detected in the lower dose cohorts, higher doses (1.5 × 10^7^ and 3 × 10^7^ PFU) elicited a dose‐dependent increase in anti‐ORFV IgG antibodies. However, neutralizing antibodies against ORFV remained undetectable, suggesting negligible vector‐specific immunity. This could facilitate repeated vaccine administration without significant anti‐vector interference, representing a substantial advantage over other viral vector‐based vaccine technologies where anti‐vector immunity prevents effective re‐immunizations [149, 150, 151, 152, 153].

### 4.3 Conclusion of the Development of Prime‐2‐CoV

The comprehensive preclinical evaluation in murine, hamster, and non‐human primate models, alongside Phase I trials (ORFEUS and UKT) in humans, has established Prime‐2‐CoV as a safe and immunogenic multi‐antigenic vaccine candidate. Co‐expression of Spike and Nucleocapsid antigens enabled broad, cross‐variant coverage, without interfering with one another. Responses were influenced by age and pre‐existing immunity, with doses below 3 × 10^5^ PFU insufficient to enhance SARS‐CoV‐2–specific responses in previously vaccinated individuals, and doses below 3 × 10^6^ PFU failing to induce ORFV‐specific antibodies in ORFV‐naïve participants. Notably, no ORFV‐neutralizing antibodies were detected, and SARS‐CoV‐2‐specific polyfunctional CD4^+^ and CD8^+^ T cells were induced at higher doses—underscoring the platform's capacity for robust cellular immunity. These findings suggest that higher doses may further augment immunogenicity, particularly in older adults, and that the absence of anti‐vector immunity supports repeated booster vaccinations. Moreover, an extension of the study with escalated doses is planned and will provide deeper insights into dose‐dependent safety and immunogenicity across different subpopulations.

**FIGURE 4:**
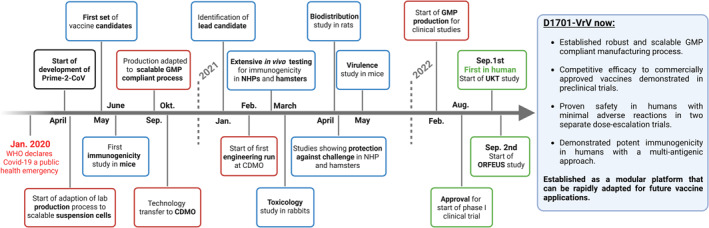
Milestones of Prime‐2‐CoV to enter clinical trials. Timeline highlighting critical milestones in the development of Prime‐2‐CoV, from the start of the project in response to the Covid‐19 pandemic to the first in human clinical trials, as well as their ultimate impact on D1701‐VrV based therapeutics. Blue borders mark the advances in the pre‐clinical development of the vaccine candidate, red the development of a corresponding GMP compliant production process and green the clinical trials.

By validating D1701‐VrV in first‐in‐human studies, Prime‐2‐CoV marks a pivotal milestone for ORFV‐based therapeutics. Figure 4 chronologically summarizes the key developmental steps of Prime‐2‐CoVs transition from laboratory research to clinical trials.

## 5 Leveraging Prime‐2‐CoV Achievements for Future ORFV‐Based Therapeutics

### 5.1 Impact on D1701‐VrV‐Based Vaccines

The D1701‐VrV platform has demonstrated remarkable versatility and adaptability, making it suitable for a wide range of infectious diseases, including those requiring robust T cell immunity, such as chronic infections and cancers [154, 155]. The experiences and progress from the development of Prime‐2‐CoV provide a solid foundation for future vaccines based on the D1701‐VrV platform, offering several key advantages that can streamline and enhance future projects.

One of the most significant benefits is the establishment of a GMP‐compliant manufacturing process for Prime‐2‐CoV. Demonstrating feasibility of D1701‐VrV‐based vaccines to be produced according to regulatory standards is of utmost importance; without this proof, the technology might have reached a dead end. This established process serves as a platform that can be readily applied to the production of new D1701‐VrV‐based vaccine candidates. Critical manufacturing parameters, quality control procedures, and documentation are already in place, facilitating efficient adaptation, scaling, and production. This reduces the time and resources required to bring additional vaccines into clinical trials.

The clinical trials of Prime‐2‐CoV have provided critical validation of the D1701‐VrV platform’s safety and immunogenicity in humans [135, 136]. The vaccine was shown to be well‐tolerated, with a favourable safety profile and minimal AEs. Importantly, it was capable of inducing robust humoural and cellular immune responses, including strong neutralizing antibody titres and T cell responses without inducing anti‐vector immunity. This clinical data serves as strong proof of concept, instilling confidence in the platform’s potential for future vaccines targeting different pathogens. Having established safety data reduces the developmental risks associated with new vaccine candidates and may expedite the early phases of clinical development.

Beyond these key points, the prior interactions and approvals with regulatory agencies for Prime‐2‐CoV have familiarized regulators with the D1701‐VrV platform. Existing data and precedents can be leveraged, reducing uncertainties and expediting the review process for future vaccines, as the platform’s characteristics, manufacturing processes and safety profile are already understood. In addition, collaborations established with research institutions, industry partners, and global health organizations can be leveraged for future vaccine development efforts. The development of Prime‐2‐CoV has also strengthened public and scientific trust in the D1701‐VrV platform, which facilitates funding acquisition from investors or through non‐dilutive funding sources.

Despite the numerous advantages, there are still challenges and gaps that need to be addressed in future D1701‐VrV vaccine endeavours. Since only a limited number of participants have been vaccinated with Prime‐2‐CoV, rare AEs might not have been recognized. Further clinical studies, especially Phase II and Phase III trials, are needed to address this point and gain more confidence regarding the safety and tolerability of the vaccine. Additionally, no commercial manufacturing scale is currently established, necessitating the scale‐up of the existing process.

Moreover, each new vaccine candidate may differ significantly from Prime‐2‐CoV, especially if modifications to the vector genome extend beyond the exchange of target antigens. Such changes could alter the vector’s properties, affecting its safety profile, immunogenicity and manufacturing characteristics. Consequently, each candidate will require careful and thorough research and development to determine the optimal risk‐benefit ratio. Factors such as antigen‐specific challenges, disease‐specific immune responses, dosing regimens, administration routes, and adjuvant formulations may need to be adapted. While the foundational knowledge gained from Prime‐2‐CoV provides a valuable starting point, these variables necessitate individualized evaluation for each new vaccine candidate.

However, with every D1701‐VrV‐based vaccine developed, our understanding of the technology will grow, making subsequent projects more straightforward. The cumulative experience will enhance our ability to anticipate and address challenges, optimize vaccine designs and improve manufacturing processes. By leveraging these advantages and proactively addressing the identified challenges, subsequent vaccine candidates can be developed more efficiently and effectively. Continued research and development are essential to fully realize the potential of the D1701‐VrV platform and ensure broad access to these vaccines.

### 5.2 Impact on Other ORFV‐Based Therapeutics

The advancements with the D1701‐VrV‐based vaccine Prime‐2‐CoV also have significant implications for other ORFV‐based therapeutics, impacting fields such as OVT and veterinary vaccines. While these other therapeutics may not benefit to the same extent, these developments offer several advantages that will positively influence these approaches.

One key benefit is the establishment of GMP‐compliant manufacturing processes for ORFV‐based vaccines. The methodologies developed provide a framework that other ORFV‐based therapeutics can adapt to meet regulatory requirements. Traditionally, many ORFV approaches rely on primary ovine cells, such as sheep skin fibroblasts, primary lamb testis [156], primary ovine turbinate (OFTu) cells [157], or OA3.T epithelial cells of sheep origin [82, 158], for virus propagation. These cells present challenges in terms of scalability, consistency and, most important, regulatory compliance. In contrast, the use of suspension HEK293 cells, a well‐established human cell line widely used in biopharmaceutical production due to its scalability and regulatory acceptance, offers significant advantages. While the susceptibility of HEK293 cells has been reported for other ORFV strains [157, 159], not all ORFV strains can effectively replicate in these cells and might require adaptation, which carries the risk of altering viral characteristics.

Advancements in downstream processing methods, purification techniques and quality control measures for effective depletion of process related impurities to comply with regulatory requirements can also be applied to improve the manufacturing processes of other ORFV‐based products. Although these downstream methods are optimized for ORFV generated in HEK293 cells, we have not observed major differences in the performance of downstream chromatography when using ORFV material generated in different cell lines such as Vero cells or OA3.T cells. Therefore, these methods might generally be applicable but may require slight adaptations and optimizations. Additionally, the progress made in regulatory interactions can benefit other ORFV‐based therapeutics. Familiarity of regulatory agencies with ORFV vectors and associated manufacturing processes can reduce uncertainties, strengthen trust in the technology and streamline the review process for future submissions involving similar technologies.

Furthermore, data obtained from preclinical and clinical trials of D1701‐VrV‐based vaccines in humans contribute to a growing body of evidence supporting the immunogenicity and safety of ORFV for medical use. Insights into the mechanisms by which D1701‐VrV vectors induce robust immune responses provide valuable information for vaccine development in both human and veterinary medicine. The demonstration of a favourable safety and reactogenicity profile, along with the absence of systemic spread or shedding, provides evidence that other ORFV therapeutics might also have a favourable safety profile. This information can be leveraged for regulatory submissions and risk assessments of other ORFV‐based therapeutics. However, it must be considered that, in contrast to most other ORFV therapeutics, D1701‐VrV is fully attenuated, replication‐deficient in vivo and does not cause clinical symptoms, even in immunocompromised hosts. This high safety margin is a substantial advantage over other ORFV‐based therapeutics, such as the strains NZ2 [160], IA82 [91], NA1/11 [79] or OV‐SY17 [82], that are not fully attenuated and may still cause infections, posing certain risks when used in clinical settings. In particular, the use of replication‐competent ORFV strains in OVT carries inherent risks of pathogenicity and viral spread, especially in immunocompromised patients, an effect that might be desired to facilitate tumour lysis but demands careful benefit‐risk assessment.

## 6 Current and Future Research on ORFV to Improve the Technology

The successful development, preclinical and clinical testing of Prime‐2‐CoV mark significant milestones for D1701‐VrV‐based vaccines specifically, and ORFV‐based therapeutics in general. Moving forward, progress in enhancing immunogenic responses and optimizing manufacturing for ORFV‐derived therapeutics demands a deeper understanding of the molecular mechanisms of ORFV replication and host‐virus interactions.

As ORFV occupies a niche position among established viral vector platforms, much of our current understanding relies on insights borrowed from its more extensively studied relative VV. However, the precise applicability of these VV‐derived findings to ORFV remains uncertain. Moreover, given ORFV's extensive immunomodulatory potential, the functions of many of its encoded proteins are still poorly characterized and often inferred only by homology with other poxviral factors.

In recent years, this knowledge gap has begun to close. Emerging research has identified previously uncharacterized virulence factors and immunomodulators, while elucidating structural mechanisms of viral assembly and cell entry [11]. Novel omics approaches, already employed to dissect virus‐host interactions in VV, are now being applied to ORFV. For instance, quantitative proteomics has been used to investigate the differentiation of murine bone marrow–derived DCs, as well as to identify antiviral mediators in goat skin fibroblasts [161, 162]. Next‐generation RNA sequencing has uncovered critical host factors for viral entry and helped compare immune responses to wild‐type versus attenuated ORFV strains [163, 164]. Likewise, a 3D organoid model of ORFV infection in human skin promises deeper insights into ORFV biology [165]. Finally, new genetic engineering technologies, such as CRISPR/Cas9, allow for faster and more precise manipulation of poxviral genomes compared to traditional homologous recombination methods. These tools accelerate not only the rapid prototyping of vaccine candidates but also the functional investigation of viral proteins [166, 167].

### 6.1 Strategies to Enhance Immunogenicity in ORFV‐Based Therapeutics

The development of Prime‐2‐CoV has allowed a thorough comparison of D1701‐VrV with other vaccine technologies and revealed that ORFV is among the most potent vaccine technologies, inducing high cellular and humoural immune responses. However, further developments could enable the vector technology to fully realize its immunogenic potential for prophylactic and oncolytic applications.

#### 6.1.1 Controlling Antigen Expression

The immune response to a vaccine antigen largely depends on both the level and timing of antigen expression [110, 168, 169]. Higher, earlier antigen expression generally correlates with stronger humoural and cellular immune responses. In the context of viral vectors like ORFV, antigen expression can be altered at the transcriptional and translational levels via multiple approaches. As ORFV replicates entirely in the cytoplasm using its own transcriptional machinery, antigen expression can be regulated through the manipulation of viral promoters. ORFV genes are expressed in a tightly regulated temporal manner, categorized into early, intermediate and late phases. Early promoters allow prompt initiation of antigen expression upon infection, mediated by factors prepackaged within the viral particle, thus maximizing immune system exposure independent of viral replication [9, 90, 101].

In VV, precisely characterized promoter motifs have led to potent synthetic constructs offering enhanced early‐phase antigen expression that translated to stronger immune responses [169, 170, 171]. For ORFV, this approach was successfully demonstrated by Joshi et al., who showed enhanced antigen expression by employing strong native early promoters identified through ORFV transcriptome analysis [156]. At the translation level, modifying the codon usage of the antigen gene to match host cell codon bias increases protein production without altering the amino acid sequence [172]. Other, less common methods to boost transgene expression include integrating regulatory elements that stabilize mRNA transcripts, thereby prolonging half‐life and supporting sustained protein synthesis. Likewise, optimized untranslated regions or polyadenylation signals can protect mRNA from degradation [173, 174]. However, poxviruses rely solely on host ribosomes and therefore, the integration of elements that might enhance the translation such as Kozak sequences, internal ribosome entry sites (IRES), despite poxviruses do not have natural IRES activity or poly‐A leaders are currently investigated in this context [175].

#### 6.1.2 Enhancing Immunogenic Properties Through Vector Modification

Beyond optimizing antigen expression, the immunogenicity of ORFV vectors can be further enhanced by modifying the viral genome to strengthen innate and adaptive immune responses. ORFV, like other poxviruses, encodes various virulence factors that modulate the host immune response to facilitate viral replication and evade the hosts immune response [14, 89, 176]. Deleting genes encoding these factors can reduce the virus’s capacity to evade the immune system, leading to enhanced immune activation. For instance, removing genes that inhibit interferon responses, NF‐kB activation, antigen presentation and APC activation may result in the induction of stronger innate immunity [24, 32, 90, 94, 159]. Another strategy is to introduce immunostimulatory genes such as cytokines, chemokines or co‐stimulatory molecules that further boost immune responses [177, 178, 179, 180, 181]. Additionally, patterns that activate innate immune sensors can promote a more robust initial immune response [182]. Such modifications can benefit not only prophylactic vaccines but also OVT. For instance, inserting genes encoding immunostimulatory cytokines (e.g., GM‐CSF, IL‐12) [178, 180, 181] can augment antigen presentation and T‐cell priming, while transgenes that express checkpoint inhibitors (e.g., anti‐PD‐1 or anti‐CTLA‐4 scFv) or co‐stimulatory ligands (e.g., CD40L) further amplify local immune responses against tumour cells [177]. In vaccine contexts, these enhancements may increase the breadth and durability of protection [177, 178, 179, 180, 181, 183], whereas in oncolytic settings, they can trigger immune activation and minimizing systemic toxicity, resulting in more robust intratumoral lymphocyte infiltration and tumour regression in otherwise immunologically ‘cold’ tumours [184, 185, 186, 187].

Nevertheless, the consequence of deleting virulence factors or introducing genes that encode immune stimulatory elements is not always predictable. Enhanced activation of the innate immune system can lead to a stronger antiviral response, limiting viral replication and antigen expression, which may diminish vaccine immunogenicity. Moreover, compromised viral replication negatively affects large‐scale vaccine production. Thus, balancing the level of immune stimulation is crucial to avoid likely negative impacts on vaccine efficacy, safety and manufacturing feasibility. While deleting virulence factors or integrating immunostimulatory elements may improve specific immune responses, these strategies also carry the risk of undesirable outcomes and require careful evaluation, rigorous testing and a deep understanding of underlying immunological mechanisms.

#### 6.1.3 Enhancing Immune Responses by Optimizing Administration Strategies

In addition to vector modification, adjusting the administration strategy presents another avenue to improve immune responses elicited by ORFV‐based therapeutics. Our investigations have shown that ORFV vectors can be administered via multiple routes—IV, IP, SC, intradermal (ID), oral and IM—each inducing immune responses of varied strengths and characteristics [101]. For instance, IM vaccination offers a balanced profile of humoural and cellular responses. Because IM vaccination is widely accepted, it may be a primary choice for prophylactic vaccines, and the current formulation aligns well with this route. However, mucosal administration (e.g., intranasal or intratracheal) might establish robust mucosal immunity [188, 189], which is crucial for neutralizing for example respiratory pathogens at their entry point via local IgA production and mucosal T‐cell activation [190]. For vaccines requiring stronger cellular immunity, our data suggest that IV administration can induce a heightened CD8^+^ T‐cell response. This route could be advantageous for therapeutic approaches, including OVT or cancer vaccines, where potent cytotoxic immunity is key to eliminate tumour cells. Additionally, intratumoral (IT) administration has emerged as a promising strategy in OVT [191]. By directly delivering the vector into the tumour microenvironment, this approach effectively concentrates the virus at the tumour site, maximizing local immune activation, enhancing viral replication within the tumour and potentially reducing systemic side effects. However, it is important to note that IT administration may not be feasible for all tumour types, particularly those that are inaccessible or metastasized [192].

Yet shifting the route of administration can affect formulation, pharmacokinetics, pharmacodynamics and biodistribution, necessitating thorough investigation. Adjusting the dose also matters, as higher doses may provoke stronger immune responses but carry increased risks of adverse effects. Finally, the timing of prime‐boost regimens is important: an appropriately timed booster can amplify and sustain memory B and T cells, leading to longer‐lasting immunity.

Moreover, combining ORFV with other platforms in heterologous prime‐boost strategies can synergistically elevate immune responses. Such effects have been observed in heterologous COVID‐19 vaccine regimens, where mixed approaches often produced stronger and more balanced immune profiles [193].

Lastly, adjuvant‐based strategies also offer a means to improve vaccine efficacy and immunogenicity. Adjuvants are vaccine components that can enhance the magnitude, breadth and durability of the immune responses [194]. Adjuvants such as aluminium salts (alum), oil‐in‐water emulsions (e.g., MF59), Toll‐like receptor agonists or other pattern recognition receptor agonists can augment and direct the immune response by recruiting and activating additional immune cells at the injection site, or by further enhancing innate activation, thus amplifying adaptive immune responses [110].

The strategies discussed—ranging from promoter engineering and genome modifications to adjustments in dosing, prime‐boost timing and route of administration—underscore the inherent flexibility of ORFV‐based therapeutics. Fine‐tuning these parameters can further enhance both immunogenicity and safety. As ongoing research deepens our understanding of ORFV's biology, the platform stands to become even more robust and broadly applicable in both preventive and therapeutic settings.

### 6.2 Enhancing Oncolytic Replication and Tumour Tropism

One key priority in OVT is increasing viral replication and tumour tropism to maximize direct tumour cell lysis [195, 196]. ORFV's intrinsic affinity for rapidly dividing cells provides a natural selectivity, yet targeted engineering can further sharpen this focus and amplify the virus's replication capacity within the tumour microenvironment.

Classical approaches such as repeated passaging in tumour cell lines may yield viral variants better adapted to the metabolic and signalling characteristics of malignant tissue [196]. Likewise, advanced passaging or targeted evolution approaches—where selective pressures are applied in relevant tumour models—can facilitate the emergence of genetic variants with enhanced tumour‐specific replication [197, 198]. Similarly, rationally designed genetic modifications—for instance, deleting viral genes that hinder replication [199, 200], or exploiting oncogenic pathways that facilitate viral entry—can accelerate viral growth and spread in cancerous lesions while sparing normal tissues. Notably, reverse genetics approaches now offer a precise means to re‐engineer the ORFV genome. By allowing targeted insertions, deletions or point mutations, reverse genetics can optimize tumour tropism and replication without compromising safety.

Recent advances in high‐throughput screening, including CRISPR‐based techniques, also enable systematic identification of viral or host factors that support or inhibit replication, guiding precise edits to optimize its oncolytic potential [201]. However, enhancing replication must be carefully balanced against safety. A virus engineered for hyper‐replication might lose some of its natural selectivity or cause excessive off‐target effects, potentially raising toxicity concerns. Moreover, excessively rapid viral growth can trigger a vigorous antiviral response and narrow the therapeutic window. Thus, the ideal oncolytic ORFV should combine robust tumour tropism and replication with regulated immune stimulation, ensuring effective tumour cell killing and immune activation without compromising patient safety or manufacturing feasibility.

Given that oncolytic ORFV is a comparatively recent addition to the field of OVT, many of these concepts derive from more established oncolytic virus platforms, for example VV. Thus far, direct evidence confirming enhanced ORFV replication through such methods remains to be demonstrated.

### 6.3 Improving ORFV Manufacturing Process

In preparation for the clinical study of Prime‐2‐CoV, we successfully adapted our existing laboratory process to a GMP‐compliant manufacturing process. This newly established platform now provides a robust basis for scaling up production of additional D1701‐VrV‐based vaccine candidates. Despite achieving high‐quality standards, several opportunities remain for process optimization to improve yields and minimize material losses.

One key area for optimization is the development of a fed‐batch or a perfusion process, which could significantly increase cell density in bioreactors. By expanding the number of cells available for infection per production run, this approach has the potential to yield higher viral titres, as demonstrated for vaccinia virus [202, 203, 204, 205, 206, 207]. Furthermore, the current upstream process lacks media supplementation or feeding strategies. Since viral replication depends entirely on host cell metabolism, metabolic screenings to identify critical nutrients could guide the development of tailored supplementation strategies to boost ORFV productivity [208, 209, 210, 211]. The HEK 293 cell line currently in use is well‐established for viral vector production and enjoy regulatory acceptance [212]. Nevertheless, further cell line development may enhance viral replication efficiency. Targeted modifications to host cell processes—including transcriptional regulation, apoptosis control and cell cycle manipulation—could create an optimized cellular environment that promotes higher viral yields [213, 214, 215, 216]. Additionally, strategies to suppress cellular antiviral defences may further enhance ORFV replication and productivity [217]. While ORFV typically exits cells via budding or cell lysis, there is potential for increasing the rate and extent of virus release by applying chemical or physical treatments after replication peaks [218].

On the downstream side, several opportunities exist to enhance overall yield and purity [219, 220]. Our current process already incorporates depth filtration to pre‐clarify the harvest by removing cell debris and aggregates, although this step is associated with notable product losses; closer coordination with upstream processes to reduce the initial impurity load may help mitigate these losses [111]. A main improvement target is the optimization of chromatography methods to boost product recovery and remove impurities such as host cell DNA (hcDNA) and host cell proteins (hcProtein). Currently, the polishing step employs Capto Core 700 resin, which has shown only moderate effectiveness. Advanced chromatography techniques—including ion‐exchange, hydrophobic interaction and multimodal chromatography (as explored by Lothert, Pagallies et al. [113])—offer potential alternatives. However, these approaches have not been extensively optimized, particularly with respect to buffer conditions. Refining buffer compositions to minimize viral aggregation and maintain particle stability could significantly improve the performance of these chromatography methods. Additionally, tangential flow filtration offers a promising strategy for both concentration and diafiltration, enhancing virus concentration and facilitating effective buffer exchange to increase the recovery of infectious particles and overall process efficiency.

A further critical limitation of the current process is its lack of adaptation to aseptic manufacturing. Given the dimensions of ORFV particles (∼160 × 260 nm), sterile filtration using a conventional 0.2 μm (200 nm) filter results in significant product loss. Transitioning to an aseptic manufacturing workflow would eliminate the need for final sterile filtration, thereby preventing product loss and improving overall yield.

Finally, formulation and stability enhancements remain a priority. According to the World Health Organization, more than 50% of vaccines worldwide are discarded due to cold chain challenges during transportation [221]. Although Prime‐2‐CoV shows excellent stability when frozen and at 4°C for over 24 months, and stability of up to 1 month at 25°C, developing a freeze‐dried (lyophilized) formulation might be desirable. Such a formulation could allow for longer storage at higher temperatures without the need for a cold chain, as demonstrated for other vaccines [222, 223, 224]. This advancement would be particularly advantageous for distribution in regions with limited refrigeration infrastructure.

In summary, improving the manufacturing process would result in several important advantages. Higher yields and more effective depletion of process contaminants would justify higher vaccine doses, which might be needed for therapeutic use like OVT or cancer vaccines. Increased production efficiency would result in more doses per manufacturing run, enabling faster provision and distribution of vaccines in emergency settings, and additionally, lower the cost per dose, making vaccines more accessible. Enhancing thermal stability through formulation improvements would allow for longer shelf life and reduced reliance on cold chains, offering logistical advantages and further reducing costs. These improvements not only benefit D1701‐VrV‐based therapeutics but might also have implications for other ORFV‐based vaccines and therapeutics, enhancing their feasibility on global health.

## 7 Future Perspective and Conclusion

The journey of ORFV‐based therapeutics from bench to bedside has been marked by significant scientific and technological breakthroughs. As detailed throughout this manuscript, ORFV exhibits robust immunomodulatory properties that have been successfully harnessed in both prophylactic and therapeutic applications. The extensive preclinical studies, coupled with early‐phase clinical evaluations, underscore the platform's versatility—not only as a vaccine vector but also as a potential oncolytic agent and immune modulator for infectious diseases.

One of the most notable achievements is the development and clinical evaluation of Prime‐2‐CoV, which has validated the safety, immunogenicity, and scalability of the D1701‐VrV platform in humans. This milestone provides a strong proof‐of‐concept that supports further exploration of ORFV‐based approaches in the broader field of infectious diseases, including the possibility of creating multivalent vaccines that can simultaneously target multiple pathogens. The ability of ORFV vectors to induce balanced immune responses—with potent activation of both humoural and cellular immunity while avoiding significant anti‐vector responses—sets them apart from many other viral platforms. This unique profile not only facilitates effective prime‐boost regimens but also supports repeated administration, an essential feature for addressing diseases that require long‐term immunosurveillance.

In parallel, ORFV's applications in veterinary medicine continue to evolve. The platform has already demonstrated its capacity to reduce disease incidence in livestock and improve vaccine efficacy in various animal models. As the technology matures, it is likely that ORFV‐based vaccines could play a crucial role in managing outbreaks of zoonotic diseases and in reducing the reliance on antibiotics—thus contributing to global efforts to combat antimicrobial resistance.

Looking ahead, several key areas warrant further research and development. First, a deeper understanding of ORFV's molecular mechanisms of immune modulation and host‐virus interactions will be essential for refining vector design. Advanced omics technologies and innovative genetic engineering tools, such as CRISPR/Cas9, offer promising avenues to dissect the roles of individual ORFV‐encoded proteins, potentially leading to the rational design of even safer and more effective vectors.

Second, enhancing immunogenicity remains an important objective. Investigating strategies to further improve immune responses includes optimizing antigen expression through genetic modifications, incorporating immunostimulatory molecules or exploring different routes of administration to elicit stronger or more targeted immune responses. For instance, adjusting promoter strength or codon optimization could enhance antigen expression levels and immunogenicity.

Third, improving manufacturing processes remains a priority. While significant progress has been made in establishing GMP‐compliant production systems, further improvements in yield, downstream processing, and formulation stability are needed to ensure that ORFV‐based products can be produced at scale and distributed efficiently worldwide. The development of lyophilized formulations, for example, could dramatically enhance the thermal stability and accessibility of these vaccines in regions with limited cold‐chain infrastructure.

Lastly, as the field moves forward, strategic combination therapies are likely to emerge as a powerful approach. The integration of ORFV‐based therapeutics with existing vaccine platforms, targeted therapies or immunomodulatory agents holds the potential for synergistic effects. Such combinations may not only enhance therapeutic efficacy but also overcome resistance mechanisms and broaden the protective scope against evolving pathogens and tumours.

In conclusion, the progress achieved in ORFV‐based therapeutics thus far offers a promising glimpse into the future of vaccine and immunotherapy development. The unique attributes of ORFV—its robust immune stimulation, favourable safety profile and flexibility in vector design—position it as a transformative technology with the potential to address critical gaps in both human and veterinary medicine. Continued multidisciplinary efforts including research and manufacturing, will be vital to fully harness the potential of ORFV, ultimately improving public health outcomes and advancing the next generation of therapeutic interventions.

## Author Contributions

M.H. and R.A. both contributed to data research, as well as to the discussion, writing, review and editing of this manuscript.

## Conflicts of Interest

R.A. holds ownership interest in Prime Vector Technologies GmbH, a company involved in the development of ORFV‐based vaccines. Additionally, R.A. is an inventor of patents related to ORFV, including a patent application of Prime‐2‐CoV_Beta (EP23730776). M.H. declares no competing interest.

## Data Availability

Figures created in BioRender. Amann, R. (2025) https://BioRender.com/t64y868.
